# Supplementary figures and images for: In silico discovery and biological validation of ligands of FAD synthase, a promising new antimicrobial target
Source: PLoS Comput Biol. 2020 Aug 14;16(8):e1007898. doi: 10.1371/journal.pcbi.1007898 (PMC7449411; doi:10.1371/journal.pcbi.1007898)

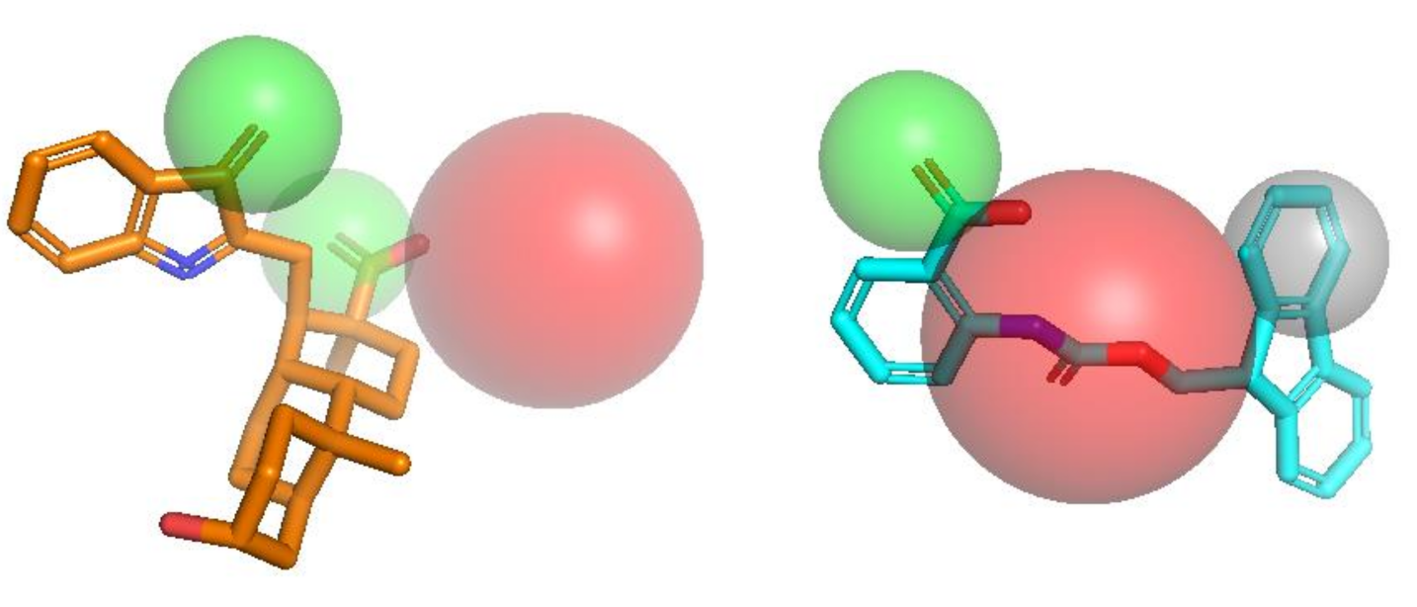

Supplement: S1 Fig — The green, red and gray spheres represent hydrogen-bond acceptor, negatively charged and aromatic features, respectively. (TIF) [file pcbi.1007898.s001.tif]

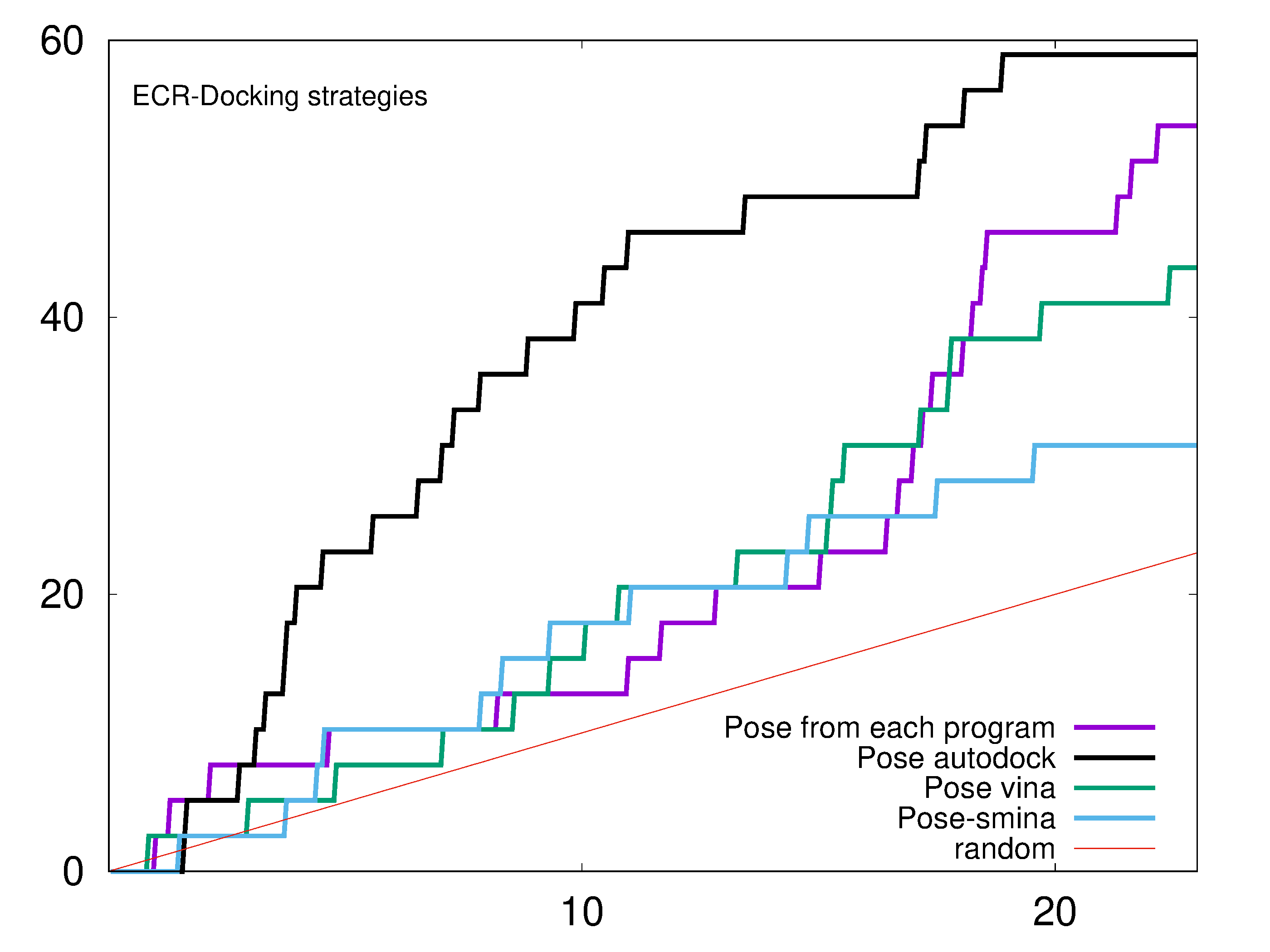

Supplement: S2 Fig — The violet line shows the EP from ECR combination of Autodock4.2, Vina and Smina docking results using the best pose from each program (as was done in ref. [43]). We also studied the outcome when using the best pose for each molecule from the different programs: from Autodock4.2 (black), Vina (green) and Smina (blue), then re-scored it with Autodock4.2, Vina, Vinardo and CYscore, and these new scores were combined using an ECR methodology. We find that using the Autodock4.2 pose and re-scoring it with the other programs produces the best outcome. (TIF) [file pcbi.1007898.s002.tif]

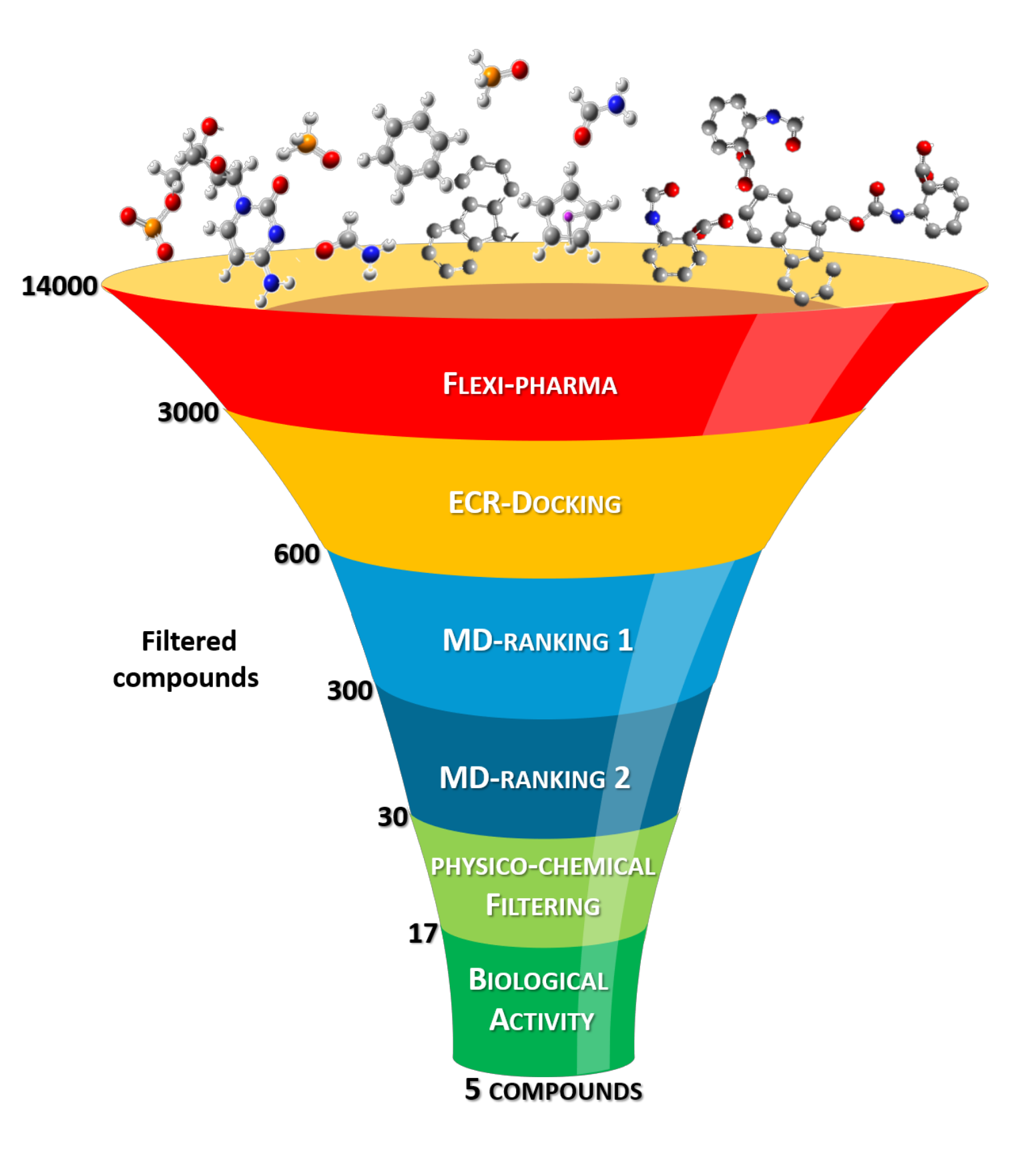

Supplement: S3 Fig — The number of filtered molecules is shown on the left. The computational protocol has several stages: first, a pharmacophore-based VS (flexi-pharma), then ECR-Docking, afterwards two MD stages (that depended on the simulation time) were used for ranking the compounds with a Morse-based score and an ECR combination of scoring functions. In the physico-chemical stage, we assessed a range of properties for the 30 best VS-ranked compounds that relate to their potential drug-likeness (S1 Table), as well as their commercial availability, selecting 17 compounds for the experimental assays. 5 compounds were found to be ligands of CaFADS. (TIF) [file pcbi.1007898.s003.tif]

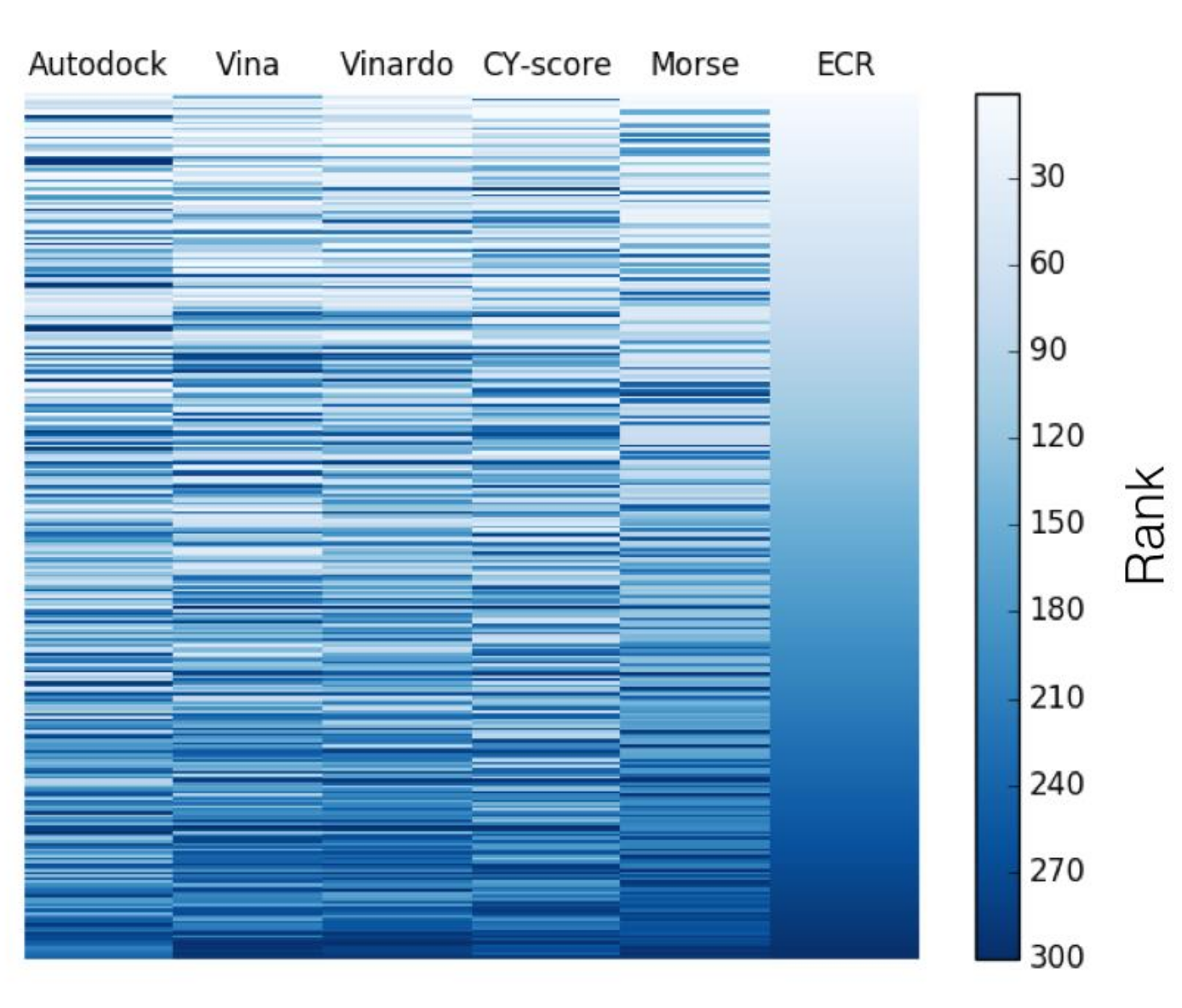

Supplement: S4 Fig — The best ranked molecules by the ECR are also well ranked for the majority of the programs but not necessarily for all. The top 30 molecules given by the ECR are selected for the following stage. (TIF) [file pcbi.1007898.s004.tif]

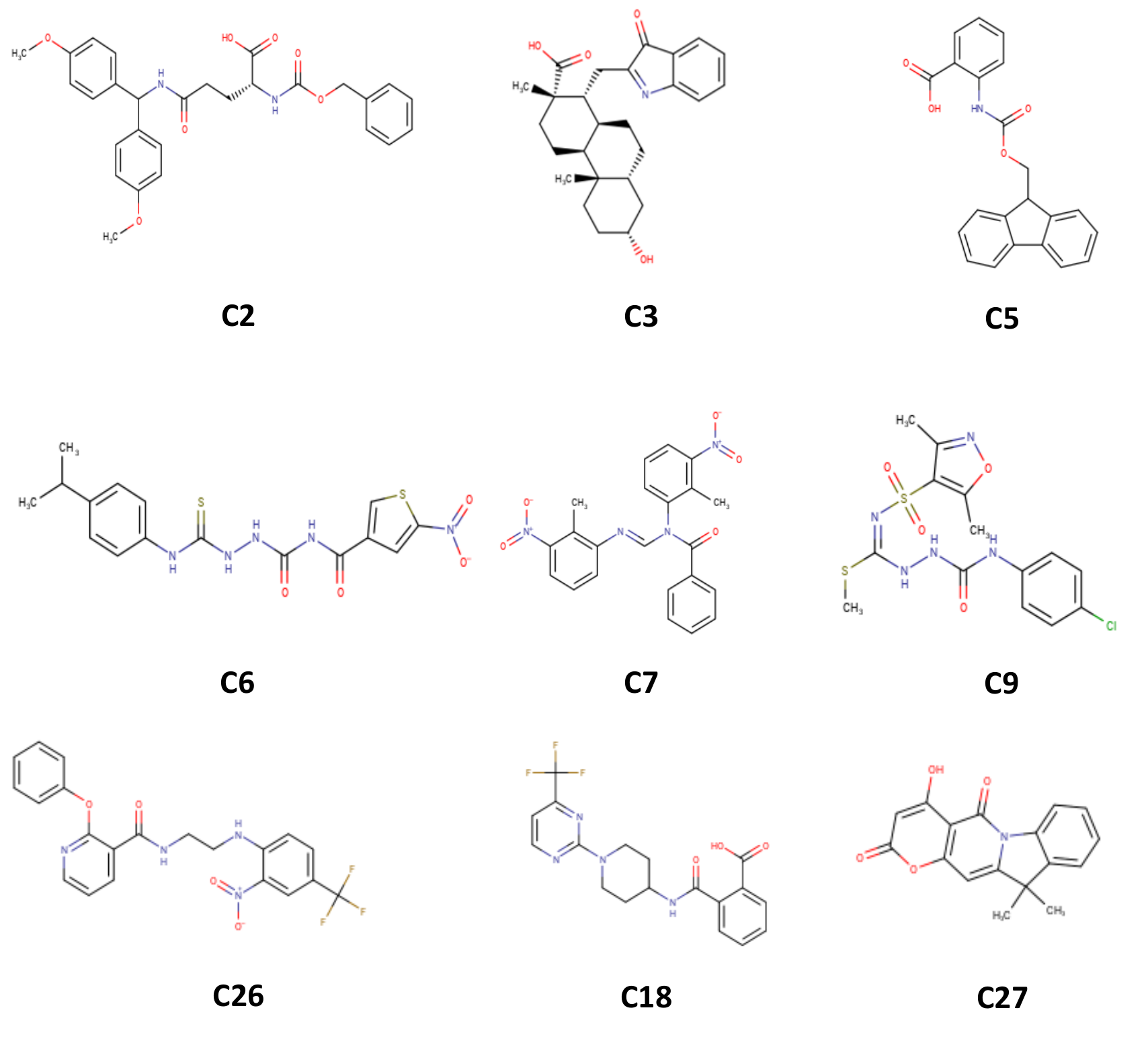

Supplement: S5 Fig — C3, C5, C6, C9 and C18 are able to bind to CaFADS. C3, C5, C6 and C18 cause 50% of FMNAT activity inhibition (IC50) at concentration lower than 250 μM. C3, C5 and C18 cause 50% RFK-FMNAT activity inhibition (IC50 at concentration lower than 250 μM. C3, C5, C6, C9, C18 and C26 have FMNAT residual activity < 95% at 250 μM of compound. C2, C3, C5, C6, C7, C9, C18 and C26 have RFK+FMNAT residual activity < 95% at 250 μM of compound. C2, C5, C6 and C27 have MIC values lower than 256 μM against C. ammoniagenes, and or C2, C3, C5, C6 and C27 Mycobacteriumspecies. (TIF) [file pcbi.1007898.s005.tif]

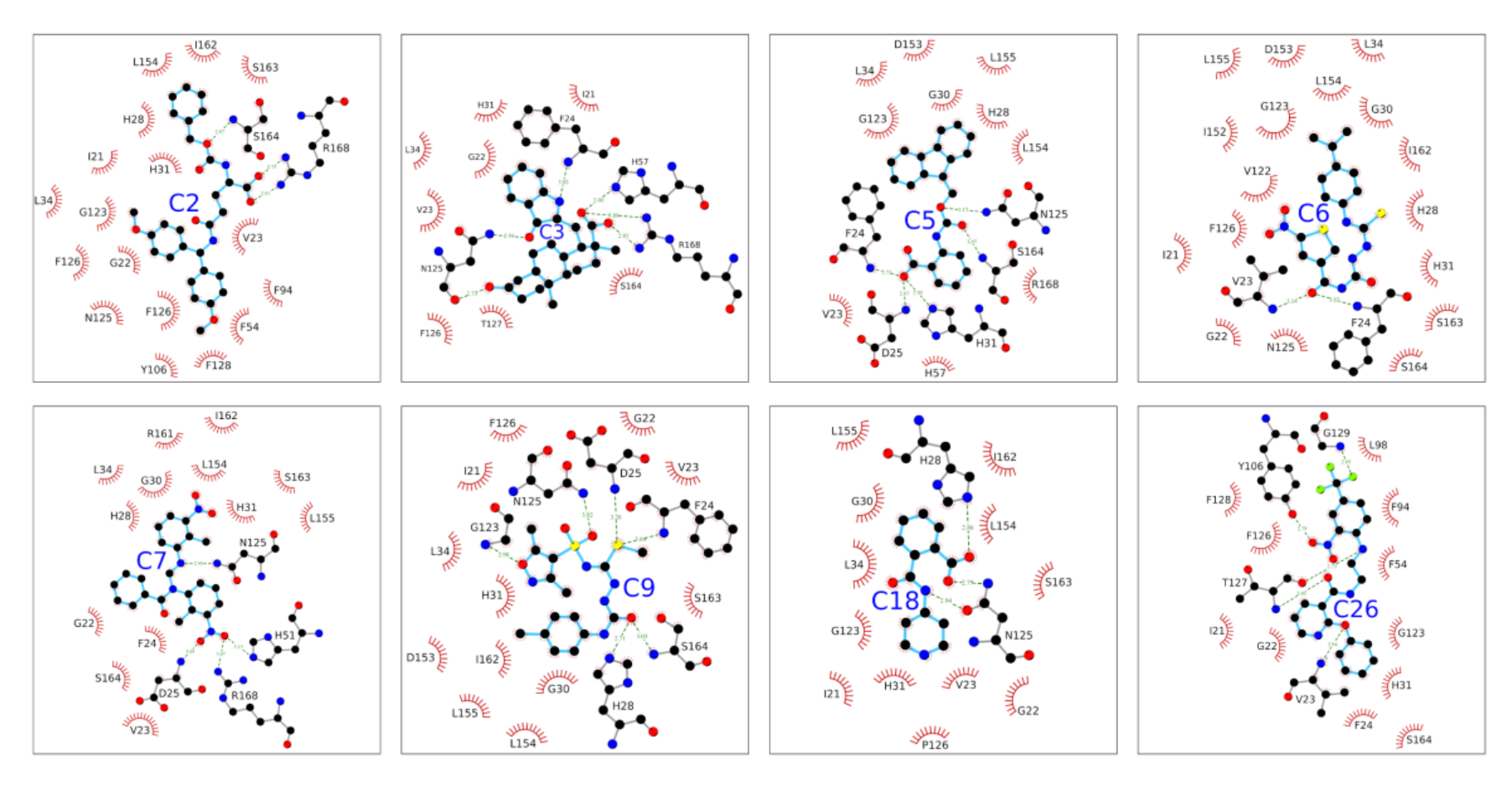

Supplement: S6 Fig — The Autodock pose obtained in the docking stage was used to observe the interactions with CaFADS. Compounds C2, C3, C5, C6, C7 and C9 interact with the ATP binding site and compounds C18 and C26 show interactions with the binding site of both ATP and FMN. Almost all compounds interact with key residues in the binding pocket: H28, H31, H57, N125, S164 and R168. (TIF) [file pcbi.1007898.s006.tif]

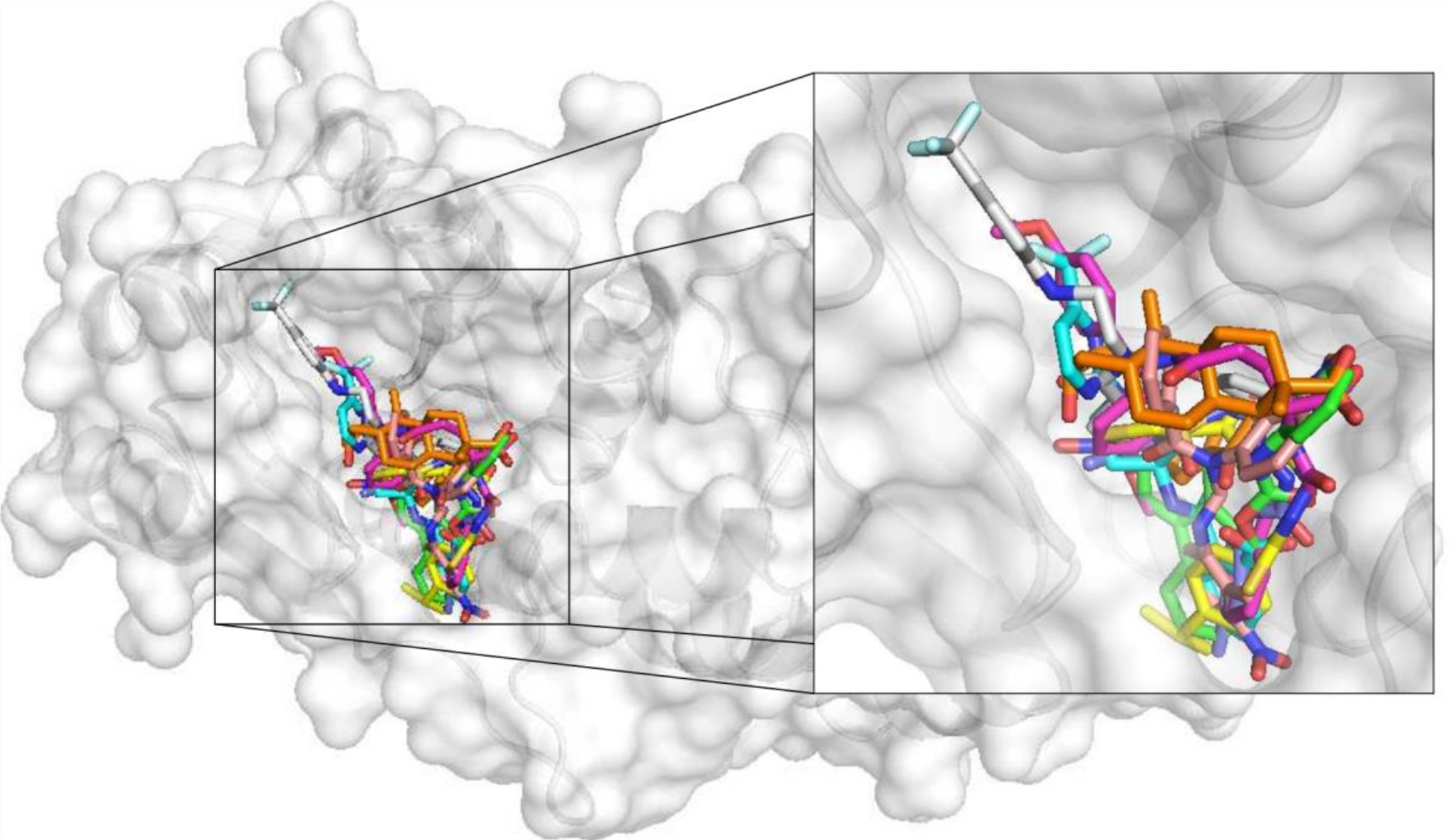

Supplement: S7 Fig — A global view of the receptor structure with the superposition of the Autodock poses of the VSHs compounds. The docked compounds cover a wide range of the receptor binding pocket. (TIF) [file pcbi.1007898.s007.tif]
