# Supplementary material for: In silico discovery and biological validation of ligands of FAD synthase, a promising new antimicrobial target
Source: PLoS Comput Biol. 2020 Aug 14;16(8):e1007898. doi: 10.1371/journal.pcbi.1007898 (PMC7449411; doi:10.1371/journal.pcbi.1007898)
Supplement: S1 Text — (PDF) [file pcbi.1007898.s008.pdf]

SI 1 Text. Description of the different ECR-docking FADS optimization strategies.

The ECR-docking was implemented using two strategies:

1) The best ranked molecules from the step 1 were docked to the FMNAT-FADS active site using the Autodock4.2 [1–3], Vina [4] and Smina [5] programs. The scores of the best poses from each docking program (evaluated with its own scoring function) were used to obtain 3 ranks (one for each docking program). The three ranks were combined using ECR to obtain a consensus rank (violet line in Figure SI 2).

2) In the second strategy, we extracted the best pose from each program and re-evaluated it with Autodock4.2 [1–3], Vina [4], Vinardo [6] and CYscore [7] scoring functions. Then, the 4 obtained ranks were combined using an ECR strategy to obtain a consensus rank (black, green and blue lines in Figure SI 2).

We found that the best EP was obtained with the second strategy using the best pose from Autodock4.2 (black line in Figure SI 2).

References

1. Morris GM, Huey R, Lindstrom W, Sanner MF, Belew RK, Goodsell DS, et al. AutoDock4 and AutoDockTools4: Automated docking with selective receptor flexibility. J Comput Chem. 2009;30: 2785–91. doi:10.1002/jcc.21256
2. Cosconati S, Forli S, Perryman AL, Harris R, Goodsell DS, Olson AJ. Virtual Screening with AutoDock: Theory and Practice. Expert Opin Drug Discov. 2010;5: 597–607. doi:10.1517/17460441.2010.484460
3. Forli S, Olson AJ. A Force Field with Discrete Displaceable Waters and Desolvation Entropy for Hydrated Ligand Docking. J Med Chem. 2012;55: 623–638. doi:10.1021/jm2005145
4. Trott O, Olson AJ. AutoDock Vina: Improving the speed and accuracy of docking with a new scoring function, efficient optimization, and multithreading. J Comput Chem. 2009;31: NA-NA. doi:10.1002/jcc.21334
5. Koes DR, Baumgartner MP, Camacho CJ. Lessons Learned in Empirical Scoring with smina from the CSAR 2011 Benchmarking Exercise. J Chem Inf Model. 2013;53: 1893–1904. doi:10.1021/ci300604z
6. Quiroga R, Villarreal MA, Ballester P, Wong M-H, Allain N, Laguerre M, et al. Vinardo: A Scoring Function Based on Autodock Vina Improves Scoring, Docking, and Virtual Screening. Sticht H, editor. PLoS One. 2016;11: e0155183. doi:10.1371/journal.pone.0155183
7. Cao Y, Li L. Improved protein-ligand binding affinity prediction by using a curvature-dependent surface-area model. Bioinformatics. 2014;30: 1674–1680. doi:10.1093/bioinformatics/btu104
