# Supplementary material for: In silico discovery and biological validation of ligands of FAD synthase, a promising new antimicrobial target
Source: PLoS Comput Biol. 2020 Aug 14;16(8):e1007898. doi: 10.1371/journal.pcbi.1007898 (PMC7449411; doi:10.1371/journal.pcbi.1007898)
Supplement: S1 Table — The table shows the VS rank, the Zinc and Maybridge Codes, the names and summary of physico-chemical criteria (values at pH 7.0) to evaluate their potential drug-likeness. Preferred criteria values are indicated on the top in green. Favorable criteria for each compound are highlighted in a green background, those in the limit are in a yellow background and those violating the criteria are in a red background. Compounds selected for experimental evaluation as virtual screening hits (VSH) are highlighted in red font in the first four columns. (PDF) [file pcbi.1007898.s009.pdf]

SI 1 Table. Summary of properties for the *Ca*FADS best ranked VS compounds. The table shows the VS rank, the Zinc and Maybridge Codes, the names and summary of physicochemical criteria (values at pH 7.0) to evaluate their potential drug-likeness. Preferred criteria values are indicated on the top in green. Favorable criteria for each compound are highlighted in a green background, those in the limit are in a yellow background, and those violating the criteria are in a red background. The 17 compounds selected for experimental evaluation as virtual screening hits (VSH) are highlighted in red font in the first four columns. [a] n.a., not available.

Rank	Rank virtual screening	Zinc code	Maybridge code	Compound name	xlogP	Molecular Weight (Da)	H-bond Donors	H-bond Acceptors	Rotatable Bonds	Polar Surface Area (Å)
					<5	<500	<5	<12	<10	<140
C1	56.16	ZINC12339395	BTB10088	6-(tert-butyl)-1,1-dimethyl-4-{[(4-nitrobenzoyl)oxy] ethanimidoyl}indane	7.0	409	0	6	6	84
C2	47.52	ZINC08626845	BTB13323	2-{[(benzyloxy)carbonyl]amino}-5-{[di(4-methoxyphenyl)methyl]amino}-5-oxopentanoic acid	4.1	505	2	9	13	126
C3	42.55	ZINC20421376	DSHS00507	7-hydroxy-2,4b-dimethyl-1-[(3-oxo-2,3-dihydro-1H-2-indolyliden)methyl]perhydro-2-phenanthrenecarboxylic acid	4.6	424	1	5	3	90

C4	36.72	ZINC00152822	HTS06058	5-oxo-3-phenyl-5-(2-quinolinylamino)pentanoic acid	1.5	333	1	5	6	82
C5	34.71	ZINC00622112	JFD02331	2-{[(9H-9-fluorenylmethoxy)carbonyl]amino}benzoic acid	5.0	358	1	5	5	78
C6	31.90	ZINC08628159	DFP00196	N-({2-[(4-isopropylanilino)carbothioyl]hydrazino}carbonyl)-5-nitrothiophene-3-carboxamide	3.0	407	4	9	7	128
C7	29.18	ZINC08647954	RJC01786	N1-(2-methyl-3-nitrophenyl)-N1-{[(2-methyl-3-nitrophenyl)imino]methyl}benzamide	5.2	418	0	9	6	124
C8	28.27	ZINC12339036	BTB02392	3-[1-cyano-2-(3-nitrophenyl)vinyl]-1-phenyl-5-(1H-pyrrol-1-yl)-1H-pyrazole-4-carbonitrile	4.2	406	0	8	5	116
C9	27.97	ZINC13464706	CD10652	N1-(4-chlorophenyl)-2-[{[(3,5-dimethylisoxazol-4-yl)sulfonyl]imino}(methylthio)methyl]hydrazine-1-carboxamide	4.3	418	3	9	6	126
C10	27.41	ZINC01027823	GK03621	N'-[(4-cyanophenyl)methylene]-1-(4-nitrophenyl)-5-(trifluoromethyl)-1H-pyrazole-	3.6	428	1	9	6	129

				4-carbohydrazide						
**C11**	26.33	ZINC04377491	S08597	5-(4-methoxyanilino)-5-oxo-3-(3,4,5-trimethoxyphenyl)pentanoic acid	0.9	402	1	8	10	106
**C12**	26.33	ZINC04381325	PD00681	6-{[4-(4-chlorophenoxy)anilino]carbonyl}-3,4-dimethylcyclohex-3-ene-1-carboxylic acid	5.5	399	1	5	5	78
**C13**	25.13	ZINC01047279	SPB00790	2-{[5-[4-(tert-butyl)phenyl]-4-(2-furylmethyl)-4H-1,2,4-triazol-3-yl]thio}-3-nitropyridine	5.8	435	0	8	7	103
**C14**	24.60	ZINC04698082	RDR00756	3-(1,3-benzodioxol-5-yl)-2-(benzoylamino)acrylic acid	2.7	310	1	6	4	87
**C15**	24.58	ZINC08636642	HTS07543	3-(4-methylphenyl)-7,9-diphenylpyrido[3',2':4,5]furo[3,2-d]pyrimidine-2,4(1H,3H)-dione	6.0	444	0	6	3	80
**C16**	23.85	ZINC03128794	BTB02953 (n.a)[a]	5-({[1-(2,4-dimethylphenyl)-1H-1,2,3,4-tetraazol-5-yl]thio}methyl)-3-(3-nitrophenyl)-1,2,4-oxadiazole	4.2	409	0	10	6	128

**C17**	23.77	ZINC01033276	HTS06059	5-[4-(aminosulfonyl)anilino]-5-oxo-3-phenylpentanoic acid	-0.13	361	3	7	7	129
**C18**	23.63	ZINC01048374	AW00987	2-[({1-[4-(trifluoromethyl)pyrimidin-2-yl]piperidin-4-yl}amino)carbonyl]benzoic acid	2.8	393	1	7	5	98
**C19**	22.38	ZINC08629386	BTB08256	1-(2-methoxyphenyl)-5,5-dimethyl-2-{[2-nitro-4-(trifluoromethyl)phenyl]thio}-1,4,5,6-tetrahydropyrimidin-4-one	5.1	453	0	7	6	88
**C20**	22.32	ZINC31723536	DSHS00186	2-(1-naphthylmethylidene)-1,2,3,4-tetrahydronaphthalen-1-one 1-(2,4-dinitrophenyl)hydrazone	8.4	464	1	8	5	116
**C21**	21.53	ZINC04381326	PD00681	6-{[4-(4-chlorophenoxy)anilino]carbonyl}-3,4-dimethylcyclohex-3-ene-1-carboxylic acid	5.5	399	1	5	5	78
**C22**	20.91	ZINC08636573	HTS06857	3,7-bis(4-methylphenyl)-2-(4-nitrophenyl)-7-oxo-5-phenyl-2,4-heptadienenitrile	7.4	498	0	5	8	86

**C23**	20.12	ZINC33380709	BLT00007	methyl 4-[2-(2,4-dinitrophenyl)hydrazono]-4-(4-methylphenyl)butanoate	5.2	386	1	10	9	142
**C24**	19.93	ZINC01042691	RF01303	[3-(2,6-dichlorophenyl)-5-methylisoxazol-4-yl]methyl 4-nitrobenzoate	5.2	407	0	7	6	98
**C25**	19.63	ZINC08618071	FM00297	[3-(2,6-dichlorophenyl)-5-methylisoxazol-4-yl]{2-[(4-methyl-2-nitrophenoxy)methyl]-1,3-thiazolan-3-yl}methanone	4.8	508	0	8	6	101
**C26**	19.61	ZINC08649294	RH01964	N-{2-[2-nitro-4-(trifluoromethyl)anilino]ethyl}-2-phenoxynicotinamide	4.2	446	2	8	9	109
**C27**	19.03	ZINC15923715	NRB05091	4-hydroxy-11,11-dimethyl-5,11-dihydro-2H-pyrano[2',3':4,5]pyrido[1,2-a]indole-2,5-dione	2.7	294	0	5	0	75
