# Supplementary material for: In silico discovery and biological validation of ligands of FAD synthase, a promising new antimicrobial target
Source: PLoS Comput Biol. 2020 Aug 14;16(8):e1007898. doi: 10.1371/journal.pcbi.1007898 (PMC7449411; doi:10.1371/journal.pcbi.1007898)
Supplement: S2 Table — Compounds were, in most cases, assayed in the 0-256 μM concentration range. In some cases, they were assayed only in the 0-64 μM concentration range, and if no effect was observed in these cases > 64 is shown. Best performing compounds are colored from red, orange, yellow to green. (PDF) [file pcbi.1007898.s010.pdf]

Table SI 2. **Minimal Inhibitory Concentration (MIC) of VSHs against different microorganisms.** Compounds were, in most cases, assayed in the 0-256 µM concentration range. In some cases, they were assayed only in the 0-64 µM concentration range, and if no effect was observed in these cases >64 is shown. Best performing compounds are colored from green, yellow, orange to red.

		Virtual Screening Hit MIC (µM)																
**Bacteria**	**Media**[a]	**C2**	**C3**	**C4**	**C5**	**C6**	**C7**	**C8**	**C9**	**C10**	**C11**	**C12**	**C14**	**C17**	**C18**	**C25**	**C26**	**C27**
	BHI	64	>64	>64	>64	>64		>64	>64	>64	>64		>64					
	MH	128	>256	>256	32	128	>256	>256	>256	>256	>256	>256	>256	>256	>256	>256	>256	128
	MH+DIP	128	>256	>256	32	128	>256	>256	>256	>256	>256	>256	>256	>256	>256	>256	>256	64
	MH+DFO	128	>256	>256	32	128	>256	>256	>256	>256	>256	>256	>256	>256	128-256	>256	>256	128
C. ammoniagenes	MH+DIP+DFO	64	>256	>256	32	128	>256	>256	>256	>256	>256	>256	>256	>256	256	>256	>256	128
C. glutamicum	BHI	16	>64	>64	32	>64	>256	>64	>64	>64	>64	>256	>64	>256	>256	>256	>256	64
	BHI	64	>64	>64	64	>64		>64	>64	>64	>64		>64					
C. diphteriae	MH	128	>256	>256	32	128	>256	>256	>256	>256	>256	>256	>256	>256	32	>256	>256	64
M. tuberculosis	7H9G-ADC	256	256	>256	128	128	>256	>256	>256	>256	>256	>256	>256	>256	>256	>256	>256	256
M. smegmatis	7H9G-ADC	256	>256	>256	>256	>256		>256	>256	>256	>256		>256					
	BHI	256	256	>256	128	>256		>256	>256	>256	>256		>256					
S. pneumoniae	MH	>256	256	>256	256	64		>256	>256	>256	>256		>256					
E. coli	LB	>64	>64	>64	>64	>64	>256	>64	>64	>64	>64	>256	>64	>256	>256	>256	>256	>256
L. monocytogenes	BHI	>64	>64	>64	>64	>64	>256	>64	>64	>64	>64	>256	>64	>256	64	>256	>256	64
P. aeruginosa	MH	>256	>256	>256	>256	>256	>256	>256	>256	>256	>256	>256	>256	>256	>256	>256	>256	>256

S. thyphimurium	MH	>256	>256	>256	>256	>256	>256	>256	>256	>256	>256	>256	>256	>256	>256	>256	>256	>256
S. aureus	MH	128	>256	>256	32-64	>256	>256	>256	>256	>256	>256	>256	>256	>256	256	>256	>256	64
Bacillus spp.	MH	128	256	>256	32	>256	>256	>256	>256	>256	>256	>256	>256	>256	256	>256	>256	32

[a]BHI, brain heart infusion broth; MH, Cation-adjusted Mueller-Hinton broth; DIP, 2,2’-bipyridine; DFO, deferoxamine; 7H9G, Middlebrook 7H9 broth supplemented with glycerol; ADC, albumin dextrose catalase supplement.
