# Supplementary material for: In silico discovery and biological validation of ligands of FAD synthase, a promising new antimicrobial target
Source: PLoS Comput Biol. 2020 Aug 14;16(8):e1007898. doi: 10.1371/journal.pcbi.1007898 (PMC7449411; doi:10.1371/journal.pcbi.1007898)
Supplement: S3 Table — (PDF) [file pcbi.1007898.s011.pdf]

Table SI 3. **Bacterial strains tested for VSH antibacterial activity.**

Bacterial strain	Features	Source
Corynebacterium ammoniagenes ATCC 7862	Reference strain	ATCC
Corynebacterium glutamicum ATCC 13032	Reference strain	ATCC
Corynebacterium diphteriae ATCC 39255	Strain lacking diphtheria toxin	ATCC
Mycobacterium tuberculosis H37Rv	Reference laboratory strain (virulent)	ATCC
Mycobacterium smegmatis mc^2155 *ATCC 700084*	High-efficiency strain for DNA transformation	ATCC
Streptococcus pneumoniae ATCC 49619	Clinical isolated	ATCC
Escherichia coli ATCC 10536	Reference strain to test bactericidal activity of disinfectants	ATCC
Listeria monocytogenes EDGe ATCC BAA-679	Clinical isolate	ATCC
Pseudomonas aeruginosa ATCC 15442	Reference strain to test bactericidal activity of disinfectants	ATCC
Salmonella thyphimurium SV5015	*Salmonella thyphimurium* SL1344 his^+	[1]
Staphylococcus aureus CECT 794 (ATCC 29213)	Reference strain to test antimicrobial susceptibility	CECT
Bacillus sp. CECT 40	Environmental isolate	CECT

ATCC, *American Type Culture Collection*
CECT, *Spanish Type Culture Collection*

References

1. Qi SY, Moir A, David O'Connor C. Proteome of Salmonella typhimurium SL1344: Identification of novel abundant cell envelope proteins and assignment to a two-dimensional reference map. J Bacteriol. 1996;178: 5032–5038. doi:10.1128/jb.178.16.5032-5038.1996
