# Supplementary material for: In silico discovery and biological validation of ligands of FAD synthase, a promising new antimicrobial target
Source: PLoS Comput Biol. 2020 Aug 14;16(8):e1007898. doi: 10.1371/journal.pcbi.1007898 (PMC7449411; doi:10.1371/journal.pcbi.1007898)
Supplement: Attachment — Submitted filename: Rebuttal.pdf [file pcbi.1007898.s012.pdf]

Response to Reviewers, manuscript PCOMPBIOL-D-20-00622

Reviewer #1: The authors propose a virtual screening cascade for unexplored targets and/or targets without co-crystallized ligands. They apply this strategy to the FAD synthase, calibrate the protocol with some known compounds and then run the workflow that includes different steps with MD simulation, rescoring, consensus scoring with a method that the group has developed; They then select some molecules coming from this protocol and test the molecules experimentally and identify several hits. This is in my opinion a very interesting study. Yet, I have several points and questions below that should be very easy to clarify but I think missing in the present version of the manuscript.

We thank the Reviewer for their comments that have helped us clarify our manuscript.

1) Open Babel uses simple rules for the protonation of essentially amino acids, which can be perfectly fine for some chemical groups on some compounds, but for many small drug-like molecules, the package fails. The authors should explain what is the protonation state of the compounds as it has obviously an impact for scoring and for MD simulation. We have on our side used extensively Open Babel and we know that the protonation is wrong for many many groups...; Thus the question.

The Maybridge and Prestwick databases, specify the protonation states of the compounds. Thus, we use the same protonation states that are reported in the database. However, during the docking calculations the hydrogen atoms that are bonded to hydrophobic carbons are deleted. We used Open Babel, in combination with a local script, to protonate back those atoms, having as template the original protonation state from the database.

Also, how do the authors deal with standardisation of the compounds? One Nitro group can be neutral, or charged; it depends the way it is written in the file.

Similarly to the protonation states, the state of the nitro group was kept as determined from the original database.

The point about protonation state of the compounds appears in the MD method section, maybe it belongs to the Docking section?

As mentioned above, for the docking programs, we used the protonation state for each molecule defined by the compound library. However, since the output poses from Autodock4.2 (used as the starting point for the MD) do not contain some protons, we had to protonate the molecules to run the MD. This has now been clarified in both sections.

2) The Flexi-Pharma method is not published but used in the present study but not all the authors of the Flexi-Pharma method are involved in this study, why (eg., Prof Cavasotto is not co-author here)?

Although Prof. Cavasotto developed the Flexi-pharma method, he did not perform any of the analysis for this current publication. For this reason, he is not an author of this paper.

However, we have now put him in the acknowledgements due to his help in developing the Flexi-pharma method.

The authors wrote in the section Flexi-pharma: First, an MD simulation of ligand-free receptor is performed. But there is no explanation of how this is performed? how long etc...; with constraints etc. I do not think this is in the supplement?

The MD conformations (used in the Flexi-pharma analysis) were taken from a previous simulation performed in Ref. 42. We used 600 equidistant frames from 60 ns of MD of *Ca*FADS. We have now introduced these details in the Methods.

With flexibility in the definition of the pharmacophore, don't the authors see an increase of noise compared to a much smaller set of pharmacophores? In docking small compounds, using pre-generated conformational ensemble, one tends to observe improvements in hit recovery only if there are not too starting conformation of the receptor. As soon as the number of receptor reaches 5 or so, there is no improvements but noise, meaning almost all compounds fit in the pocket. Is it the situation here for the definition of the pharmacophore models?

Indeed, with the Flexi-pharma method, tens of thousands of pharmacophores might be generated from all conformations. However, from our benchmarking analysis, we have not found an increase in the noise due to the large amount of pharmacophores. We now tested the Flexi-pharma method over twenty systems, and we find a good performance for 19 (see section 3.2 in ref. 31 and the SI). A possible reason for why the large set of pharmacophores does not hinder the outcome is because of the voting scheme, where we only assign one vote per conformation instead of counting the large number of, possibly, matched-pharmacophores.

3) With regard to molecular docking, the authors should explain the type of compound input files that they used for Smina (a single mol2 or sdf file ? or pdbqt?) as this can change significantly the scores for some molecules.

All docking programs used the same format for the input molecules, which was pdbqt. These files were created using the information (protonation states, etc) from the compound libraries. This has been clarified in the text.

4) The authors should show some docked poses, show and explain the properties of the binding pocket (large or small cavity, ions, cofactor or not...;).

The docking poses for the hit compounds are shown now in Fig. 7 (see also point 3 of Reviewer 3).

5) Several compounds have a nitro group (or two, this is usually not very welcome in a drug discovery project but tolerated at the hit stage) or somewhat a negatively charge group in the ligand. What is the rational with regard to the binding pocket?

The FMNAT binding pocket contains a positively charged motif (161-168), which includes residues R161 and R168. R168 is a highly conserved residue, and mutational studies suggest a key role for R161 and R168 in ATP binding for stabilizing the ATP phosphate group [42, 50]. The mutation of residue R161 produces deleterious effects in the FMNAT activity, causing an incorrect accommodation of the substrates in the active site for catalysis [50]. Several of the virtual screening hits (VSHs) interact with these positively charged residues (see also point 3 reviewer 3).

If this charge or interaction is that important, could it be the key reason of the success of the MD and/or pharmacophore and/or scoring? In a more hydrophobic pocket without this interaction, i assume maybe there is a key salt-bridge, the situation could be very different and what is happening then with the screening protocol? The authors should explain how much this approach is likely to generalise to other types of pocket.

Our main goal for the Flexi-pharma [31] and ECR docking [43] methodologies was to develop general frameworks applicable for diverse biomolecular systems in the different stages of VS. These methods were tested over multiple benchmark systems, having diverse interactions in the binding site. However, the entire protocol that includes the MD screening (funnel shown in Fig. 1) has only been optimized (with the Prestwick library) and tested (with the Maybridge library) for the FADS. For this reason, we included in the conclusions the following phrase: “Although our final results show a good experimental enrichment, we note that the generalization of the entire protocol (shown in Figure 1) for multiple systems, with diverse active sites, is still to be optimized, extensively tested and validated.”

Reviewer #2: The work described in this article is scientifically sound and looks carefully done in all its parts. Due to my specific expertise, my comments are limited and focused on the experimental part of the study for which I have no particular criticism. The article is written in a concise and and very clear style. I just recommend the Authors to carefully check and fix some minor formal typos such as the lack of a space between numbers and deg C; unit.

We thank the Reviewer for their encouraging comments. We have added the spaces and corrected the typos.

Reviewer #3: The article describing the computational approach that led the authors identify FADS inhibitor. Here authors developed new screening protocol and successfully employed to identify hit compounds in µM range that has potential to become anti-microbial therapy. The manuscript carefully written and presented. The protocol differ from classical virtual screening, where the last filter is usually docking. Here, authors exercised molecular dynamics to pick 17 compounds. Selecting the compounds include state-of-the-art physicochemical properties into consideration that increase drug-likeness.
I have some of the following concerns (minor) to be addressed before acceptance.

We thank the Reviewer for their comments and corrections that have improved our work.

1) Flexi-pharma mapping on the screened compounds are not shown.

We have included in SI 1 Fig., some examples of the pharmacophore mapping.

2) Although there is a reference to ECR method, I don't see some details in the methods as well as the ECR versus the individual scoring function. It helps to see them side-by-side in a table.

In SI 4 Fig., we compare the individual ranks from each scoring function to the ECR. As expected, the best ranked molecules by the ECR are -on average- also highly ranked for the majority of the programs. However, an advantage is that the ECR method acts as a conditional "or", and does not require that a molecule performs well for all programs.

3) Hit molecule interaction in the receptor context is not available, I will recommend to present that figure.

The VSHs interactions with the receptor are now shown in Fig. 7, SI 6 and SI 7 Figs. We have also included a new paragraph at the end of the results describing the main interactions.
